# Role of LRP1 and ERK and cAMP Signaling Pathways in Lactoferrin-Induced Lipolysis in Mature Rat Adipocytes

**DOI:** 10.1371/journal.pone.0141378

**Published:** 2015-10-27

**Authors:** Keiko Ikoma-Seki, Kanae Nakamura, Satoru Morishita, Tomoji Ono, Keikichi Sugiyama, Hoyoku Nishino, Hisashi Hirano, Michiaki Murakoshi

**Affiliations:** 1 Research and Development Headquarters, Lion Corporation, Kanagawa, Japan; 2 Advanced Medical Research Center, Yokohama City University, Kanagawa, Japan; 3 Kyoto Prefectural University of Medicine, Kyoto, Japan; 4 Ritsumeikan University, Shiga, Japan; The University of Queensland, AUSTRALIA

## Abstract

Lactoferrin (LF) is a multifunctional glycoprotein present in milk. A clinical study showed that enteric-coated bovine LF tablets decrease visceral fat accumulation. Furthermore, animal studies revealed that ingested LF is partially delivered to mesenteric fat, and *in vitro* studies showed that LF promotes lipolysis in mature adipocytes. The aim of the present study was to determine the mechanism underlying the induction of lipolysis in mature adipocytes that is induced by LF. To address this question, we used proteomics techniques to analyze protein expression profiles. Mature adipocytes from primary cultures of rat mesenteric fat were collected at various times after exposure to LF. Proteomic analysis revealed that the expression levels of hormone-sensitive lipase (HSL), which catalyzes the rate-limiting step of lipolysis, were upregulated and that HSL was activated by protein kinase A within 15 min after the cells were treated with LF. We previously reported that LF increases the intracellular concentration of cyclic adenosine monophosphate (cAMP), suggesting that LF activates the cAMP signaling pathway. In this study, we show that the expression level and the activity of the components of the extracellular signal-regulated kinase (ERK) signaling pathway were upregulated. Moreover, LF increased the activity of the transcription factor cAMP response element binding protein (CREB), which acts downstream in the cAMP and ERK signaling pathways and regulates the expression levels of adenylyl cyclase and HSL. Moreover, silencing of the putative LF receptor low-density lipoprotein receptor-related protein 1 (LRP1) attenuated lipolysis in LF-treated adipocytes. These results suggest that LF promoted lipolysis in mature adipocytes by regulating the expression levels of proteins involved in lipolysis through controlling the activity of cAMP/ERK signaling pathways via LRP1.

## Introduction

Metabolic syndrome comprises disorders that increase the risk of developing cardiovascular disease and other chronic ailments. Dietary excess, lack of exercise, and high levels of psychological stress have caused a rapid increase in the incidence of metabolic syndrome worldwide [1]. Visceral obesity is central to the development of metabolic syndrome. Excessive visceral fat accumulation disrupts the production of adiponectin, plasminogen activator inhibitor type 1, tumor necrosis factor, and nonesterified fatty acids, leading to high blood glucose levels, induction of insulin resistance, high blood pressure, and dyslipidemia [2–4]. To prevent the progression of the metabolic syndrome, lifestyle habits must be altered to achieve a balance between energy intake and consumption. Moreover, the use of certain nutrients as beneficial dietary supplements is gaining increasing attention [5].

Lactoferrin (LF) is an iron-binding glycoprotein present at high concentrations in breast milk. This protein possesses antibacterial, antiviral, immunostimulatory, antioxidative, and cancer-preventive activities [6–10]. Commercially available LF, which is produced from bovine milk, is an approved food additive in Japan and is included in the generally recognized as safe category in the United States. Moreover, LF is added to infant formula, yogurt, skimmed milk, and nutraceuticals. We initially focused on the antibacterial activities of LF and found that it potently ameliorates periodontal disease in animals [11]. Furthermore, a reduction in visceral fat levels was detected, leading to the identification of LF as a potential treatment for the metabolic syndrome.

We conducted clinical trials to determine whether dietary supplementation with LF decreases visceral fat levels and ameliorates the metabolic syndrome. Using enteric-coated LF tablets that are not degraded in the stomach, we found that treatment for 8 weeks decreased abdominal fat, particularly visceral fat, accumulation in Japanese men and women with abdominal obesity [12]. Moreover, the results of animal and *in vitro* studies support these findings [13–15]. For example, Shia et al. (2012) reported the benefits of LF for reducing body weight and fat content using diet-induced obese mice [16]. To determine the mechanism of action of orally administered LF, we determined its distribution in rat tissues [11] and detected relatively high levels of LF in mesenteric fat tissue, suggesting that LF acts directly on adipocytes. When we determined the activities of LF in preadipocytes derived from rat mesenteric fat tissue, we found that LF inhibits adipogenic differentiation as a function of its dose. However, treatment with pepsin abrogated this activity, indicating the requirement for enteric-coated LF to maximize its delivery to adipocytes [11].

The balance between lipid synthesis and degradation determines the lipid content of adipocytes. Although we found that LF inhibits lipid synthesis, to the best of our knowledge, there are no reports describing the lipolytic action of LF. Therefore, we determined the lipolytic potential of LF using mature adipocytes derived from rat mesenteric fat tissue and found that LF promoted lipolysis in a dose dependent manner. Subsequent DNA microarray analysis and determination of increased intracellular cAMP levels showed the possibility of activating cAMP signaling pathway by LF [17]. However, further studies are required to determine the functional significance of those changes, particularly in levels of transcription. To address this question, in the present study, we determined the levels of protein expression and tried to clarify the signaling pathway that mediates lipolytic action by LF in mature adipocytes. Here we report that LF activates cAMP and extracellular signal-regulated kinase (ERK) signaling pathways by increasing phosphorylation levels of key molecules, and upregulates the expression level of enzymes involved in lipolysis downstream of these pathways. Furthermore, we tried to identify the receptor, important for LF induced lipolysis in mature adipocytes. Silencing of low-density lipoprotein receptor-related protein 1 (LRP1, also known as CD91) in mature adipocytes inhibits the activation of HSL and undergoes lipolysis by LF treatment. Our findings provide new insights into the mechanism underlying the lipolytic activity of LF.

## Materials and Methods

### Materials

Bovine LF was purchased from Friesland Campina Domo (The Netherlands). Typical protein purity was 98%, according to the manufacturer’s data.

### Ethics statement

#### Animal care

All animal experiments were carried out according to protocols approved by the Institutional Animal Care and Use Committee of Lion Corporation, Japan. All surgeries were performed under anesthesia, and all efforts were made to minimize animal suffering.

### Animals and diets

Male Sprague–Dawley rats (7-weeks-old) were purchased from Japan SLC (Shizuoka, Japan) and were maintained in a barrier room at 23°C with a 12-h light/dark cycle (lights on 07:00 h–19:00 h). The animals were fed CE-2 laboratory chow (Clea Japan, Tokyo, Japan) and tap water *ad libitum*.

### Preparation of mature adipocytes

Mature adipocytes were isolated from primary cell cultures of mesenteric fat-derived preadipocytes as previously described [11]. Using a calibrated anesthetic delivery machine, Sprague–Dawley rats were induced into anesthesia at a dose of 5% isoflurane, and then maintained at a surgical plane by continuous inhalation of 3% isoflurane. Anesthetized rats were euthanized by exsanguination from the abdominal aorta. The extirpated mesenteric fat tissue was added to phosphate-buffered saline (PBS) containing 1 mg/ml of collagenase from *Clostridium histolyticum* Type IV (C5138, lot SLBC5970V, Sigma–Aldrich Japan, Tokyo, Japan) and incubated at 37°C for 40 min. The digested tissue was filtered through a 100-μm mesh (REF 352360, BD Falcon, USA), and Dulbecco’s Modified Eagle’s Medium (DMEM) was added before centrifuging the digests at 800 rpm for 10 min. The sediment was collected, washed twice with DMEM, filtered through a 70-μm mesh (REF 352350, BD Falcon, USA), and centrifuged again. The cell pellet was suspended in visceral fat differentiation medium (VACMR, Primary Cell Co., Hokkaido, Japan), and the cells were seeded at a density of 6.2 × 10^4^ cells/cm^2^ in 6-well (for proteomics analysis) or 24-well (for other experiments) plastic culture plates (Sumitomo Bakelite Co., Tokyo, Japan) coated with Cellmatrix type I-C collagen (Nitta Gelatin, Tokyo, Japan). Mature adipocytes were prepared by culturing the cells in visceral fat differentiation medium according to the manufacturer’s protocol in a humidified atmosphere containing 5% CO_2_ at 37°C for 7 days.

### Preparation of cell extracts for proteomic analysis

Cells were harvested at 1, 3, and 9 h after the addition of 1 mg/ml of LF, washed twice with ice-cold PBS, scraped into lysis buffer [8 M urea (821519, Wako Pure Chemical Industries, Ltd., Osaka, Japan), 5% sodium deoxycholate (190–08313, Wako Pure Chemical Industries, Ltd., Osaka, Japan), 0.5% Phosphatase Inhibitor Cocktail 1 (P2850, Sigma-Aldrich Japan, Tokyo, Japan), 0.5% phosphatase inhibitor cocktail 2 (P5726, Sigma-Aldrich Japan, Tokyo, Japan), 1% protease inhibitor cocktail (03969–21, Nacalai Tesque, Kanagawa, Japan), and 100 mM TEAB (pH = 7.5)], and then sonicated on ice. After centrifugation for 20 min at 15,000 rpm at 4°C, the supernatants were collected, and protein concentrations were measured using the BCA protein assay reagent kit (#23250, Thermo Scientific Japan, Kanagawa, Japan).

### Preparation of peptide samples for proteomic analysis

Protein (10 μg) extracted from mature adipocytes was digested as follows: Disulfide bonds were reduced with 10 mM (final concentration) of dithiothreitol (DTT) for 30 min at 60°C and alkylated with iodoacetamide (final concentration 25 mM) for 15 min at room temperature in the dark. After diluting the urea to 2 M with 50 mM NH_4_HCO_3_, sequencing-grade trypsin was added (1:40, enzyme:total protein) and incubated at 37°C overnight. Trypsin was inhibited with 1.0% TFA, and the solution was centrifuged at 15,000 rpm for 10 min. The supernatant was collected, and residual sodium deoxycholate was removed with ethyl acetate. After mixing and removing the upper layer, the solution was dried for approximately 5 min using a Speed-Vac to volatilize residual ethyl acetate. Peptide solutions were desalted using a self-packed SDB/C_18_ tip column (Stage tip). Stage tips were prepared by packing Empore SDB XD (5010–30046, 3M, Tokyo, Japan) and Empore C18 (5010–30006, 3M, Tokyo, Japan) column packing materials into a 200-μl pipet tip described by Rappsilber *et al*. [18]. The columns were washed with 100 μl of B buffer (80% acetonitrile (ACN)/0.1% TFA) by centrifuging at 2,000×*g* for 1 min and then equilibrated with 100 μl of A buffer (2% ACN/0.1% TFA) by centrifuging at 2,000×*g* for 1 min. Samples were centrifugally loaded at 800×*g* for 5 min, and the flow-through samples were reloaded, centrifuged, washed twice with 100 μl of A buffer, and eluted sequentially with 50 μl of 40% ACN/0.1% TFA and 50 μl of B buffer. Eluted samples were dried using a Speed-Vac and stored at −20°C.

### Proteomic analysis using shotgun LC–MS/MS

LC–MS/MS analysis was performed using an LTQ Orbitrap Velos hybrid-mass spectrometer (Thermo Fisher Scientific, Bremen, Germany) and Xcalibur version 2.0.7 software provided with the UltiMate 3000 LC system (Dionex, LC Packings, Sunnyvale, CA, USA). Relative quantitation of unlabeled proteins was performed using Progenesis LC–MS data analysis software (version 4.1, Nonlinear Dynamics, Durham, NC, USA). To identify peptides, peak lists were created using a Progenesis LC–MS and we used MASCOT (v2.4.1, Matrix Science, London, UK) to search *Rattus norvegicus* protein sequences in the UniProt Knowledgebase (UniProtKB/Swiss-Prot) database (version May 2013; 7,853 entries). The search parameters were as follows: trypsin digestion with two missed cleavages permitted; variable modifications, protein N-terminal acetylation, oxidation of methionine, carbamidomethylation of cysteine, N-terminal carbamylation, and phosphorylation of serine, threonine, and tyrosine; peptide-mass tolerance for MS data, ±5 ppm and fragment mass tolerance, ±0.5 Da. The estimation of false discovery rate (FDR) was accomplished using a MASCOT decoy database. We used 1% FDR as the cutoff to export results from the analysis, and peptides that yielded a peptide ion score ≥25 were considered as identified.

### Western blot analysis

For western blot analysis, cells were lysed in a cell extraction buffer containing 20 mM MOPS, 50 mM β-glycerophosphate, 50 mM sodium fluoride, 1 mM sodium vanadate, 2 mM EDTA, 1% NP-40, 1 mM DTT, 1 mM benzamidine, 0.5% phosphatase inhibitor cocktail 1 (P2850, Sigma-Aldrich Japan, Tokyo, Japan), 0.5% phosphatase inhibitor cocktail 2 (P5726, Sigma-Aldrich Japan, Tokyo, Japan), and 1% protease inhibitor cocktail (03969–21, Nacalai Tesque, Kanagawa, Japan). Aliquots of the lysates were resolved by electrophoresis through 4%–12% polyacrylamide gels (NP0336BOX, NuPAGE Pre-Cast Protein Gels, Life Technologies Japan, Tokyo, Japan) and blotted onto PVDF membranes. The antibodies were used in this study were as follows: anti-adenylyl cyclase 2 (AC2) (C-20) (sc-587, Cosmo Bio Co., Ltd., Tokyo, Japan), anti-AC1 (ab38331, Abcam plc, Cambridge, UK), anti-AC6 (ab14781, Abcam plc, Cambridge, UK), anti-HSL/LIPE (ab45422, Abcam plc, Cambridge, UK), anti-phospho-HSL (Ser660) (#4126, Cell Signaling Technology Japan, K.K., Tokyo, Japan), anti-c-Raf (#9422, Cell Signaling Technology Japan, K.K., Tokyo, Japan), anti-phospho-perilipin (Ser497) (4855, VALA Sciences Inc, California, USA), anti-CREB (48H2) (#9197, Cell Signaling Technology Japan, K.K., Tokyo, Japan), anti-phospho-CREB (Ser133) (87G3) (#9198, Cell Signaling Technology Japan, K.K., Tokyo, Japan), anti-LRP1(85kDa) (ab92544, Abcam plc, Cambridge, UK), and HRP-conjugated goat anti-rabbit IgG (H+L) (115-035-062, Jackson ImmunoResearch Laboratories Inc., Pennsylvania, USA). The actin band, which served as the loading control, was detected using an anti-β-actin (13E5) rabbit monoclonal antibody (#4970, Cell Signaling Technology Japan, K.K., Tokyo, Japan). ECL Prime Western Blotting Detection Reagent (RPN2232, GE Healthcare Japan, Tokyo, Japan) was used to detect antigen–antibody complexes.

### Detection of protein activity

The enzymatic activity of protein kinase A (PKA) was detected using a PKA kinase activity kit (ADI-EKS-390A, Cosmo Bio Co., Ltd., Tokyo, Japan). Phosphorylation levels of extracellular signal-regulated kinase (ERK1/2) were detected using PhosphoTracer ERK1/2 (pY202/Y204) + total ERK ELISA kit (ab119659, Abcam plc, Cambridge, UK). Active-Ras protein pull down and detection assays were conducted using an Active Ras Pull-Down and Detection Kit (16117, Thermo Fisher Scientific, K.K., Kanagawa, Japan). All assays were performed according to the manufacturers’ protocols.

### LRP1 gene silencing

Silencing of LRP1 gene expression in adipocytes was achieved using siRNA. Duplexes of 21-nucleotide long rat LRP1 siRNA (target sequence CAUCAAGCGUGGAUGCAAAtt, Silencer Select siRNA, ID#: s152487) and negative control siRNA were purchased from Applied Biosystems (Life Technologies Japan, Tokyo, Japan). Rat preadipocytes prepared as aforementioned were seeded in visceral fat differentiation medium (VACMR, Primary Cell Co., Hokkaido, Japan) at a density of 4.1 × 10^4^ cells/cm^2^ in 24-well plastic culture plates (Sumitomo Bakelite Co., Tokyo, Japan) coated with Cellmatrix type I-C collagen (Nitta Gelatin, Tokyo, Japan) and cultured for 3 days in visceral fat differentiation medium, and then were transfected with siRNA (5 nM) using INTERFERin siRNA Transfection Reagent (409–10, Polyplus transfection, Illkirch, France) according to the manufacturer’s protocol. After 66 h, the medium was replaced with fresh visceral fat differentiation medium. After 24 h (day 7), differentiated mature adipocytes were used for protein analyses. The efficiency of LRP1 knockdown determined using western blotting analysis was 75%–80%.

### Lipolysis assay of mature adipocytes

Seven days after differentiating the preadipocytes to mature adipocytes, the medium was replaced with fresh visceral fat differentiation medium (VACMR, Primary Cell Co., Hokkaido, Japan), and 1 mg/ml of LF or 0.1 mM of isoproterenol (ISO) (positive control for β1 and 2 adrenalin receptor agonist, 091–02511, Wako Pure Chemical Industries, Ltd., Osaka, Japan) was added to the medium. Untreated cells served as a control. Culture supernatants were collected for lipolysis assays 24 h after LF or ISO was added to the cultures. The PKA signal inhibition assay was performed with or without 40 mM of H-89, dihydrochloride (#9844, Cell Signaling Technology Japan, K.K., Tokyo, Japan) in the assay medium described above. Cells were treated with H-89 starting 30 min before the addition of LF. The β-adrenergic receptor inhibition assay was performed with or without 20 mM of atenolol (A413, Sigma-Aldrich Japan, K.K., Tokyo, Japan) in the assay medium described above. Cells were treated with atenorol starting at 2h before the addition of LF or ISO. Lipolytic activity was analyzed by measuring the glycerol concentration [19] in the medium using an assay kit (148270 F-kit, Roche Diagnostics, K.K., Tokyo, Japan) according to the manufacturer’s protocol.

### Statistical analysis

Data are presented as the mean ± standard deviation (SD). Proteomic analysis data were analyzed using one-way analysis of variance and the Student *t* test. Western blot data and glycerol quantitation data were analyzed using Dunnett’s test and the Student *t* test. Statistical significance was defined as *p* < 0.05, and data were analyzed using JMP version 10.0.2 software (SAS Institute, Cary, North Carolina, USA).

## Results

### Proteomic analysis of LF-treated adipocytes

Mature adipocytes were prepared from control and LF-treated groups. Each group’s samples were digested with trypsin, and the tryptic peptides (1 μg) from three samples (1, 3, and 9 h after addition of LF) were analyzed using LC–MS/MS. Each sample was prepared in triplicate and analyzed twice, and 36 samples for MS/MS analysis were processed. The raw MS/MS data were processed and we used MASCOT to query the UniProt Knowledgebase (UniProtKB/Swiss-Prot) rat database. We identified 1,285 proteins, and the details about protein identification are described in experimental procedures. The numbers of proteins that exhibited a significant change in expression levels compared with control groups at each time point [one-way ANOVA and Student *t* test (*p* < 0.05)] were as follows: 1 h, 70; 3 h, 78; and 9 h, 137. Protein expression data of the LF-treated groups (1, 3, and 9 h) were then calculated as the average fold-change compared with the control groups.

HSL, which is involved in the cAMP signaling pathway, was upregulated in response to treatment with LF. Moreover, there were important changes in the protein expression levels of components of the ERK signaling pathway, such as MAPK3 (ERK1) and Ras-related proteins. Further, we detected alterations in the expression profiles of proteins involved in lipid metabolism and in the TCA cycle (Table 1). Therefore, these data suggest that LF activates the standard lipolytic pathway (cAMP signaling pathway), and activates the ERK signaling pathway. To verify this assumption, we conducted a series of experiments that are described next.

**Table 1:** Proteins detected that are involved in signaling or metabolic pathways.

Function	Accession	Peptide count	Peptides used for quantitation	Confidence score	Anova (p)*	Description	Gene name	Trivial name	1h Fold change	p-value	3h Fold change	p-value	9h Fold change	p-value
cAMP signaling pathway														
	P15304	18	17	788.35	0.0049**	Hormone-sensitive lipase	Lipe	HSL	0.97	0.17	1.06	0.37	1.19	0.023*
	P43884	17	17	1396.82	0.0046**	Perilipin-1	Plin1	PLIN	0.88	0.032*	0.96	0.35	0.98	0.71
	P27791	1	1	56.66	0.25	cAMP-dependent protein kinase catalytic subunit alpha	Prkaca	PKA	0.88	0.58	1.71	0.082	1.39	0.41
	P12369	11	8	699.96	0.23	cAMP-dependent protein kinase type II-beta regulatory subunit	Prkar2b		1.03	0.65	1.02	0.70	1.07	0.12
	P09456	1	1	48.84	0.57	cAMP-dependent protein kinase type I-alpha regulatory subunit	Prkar1a		1.30	0.60	1.71	0.23	1.84	0.066
	P12368	4	1	302.54	0.68	cAMP-dependent protein kinase type II-alpha regulatory subunit	Prkar2a		1.07	0.86	1.42	0.21	1.67	0.18
ERK signaling pathway														
	P63086	4	2	208.46	0.62	Mitogen-activated protein kinase 1	Mapk1	ERK2	1.15	0.49	1.27	0.15	1.15	0.35
	P21708	3	2	202.13	0.21	Mitogen-activated protein kinase 3	Mapk3	ERK1	1.02	0.85	1.19	0.037*	1.18	0.19
	Q01986	4	4	113.46	0.40	Dual specificity mitogen-activated protein kinase kinase 1	Map2k1	MEK	1.06	0.74	0.79	0.078	1.01	0.89
	O35509	6	6	300.16	0.0069**	Ras-related protein Rab-11B	Rab11b	Ras protein	1.01	0.90	1.20	0.024*	1.00	0.90
	P35281	4	2	221.96	0.29	Ras-related protein Rab-10	Rab10		0.92	0.73	1.24	0.42	1.74	0.023*
	P70550	4	2	223.99	0.23	Ras-related protein Rab-8B	Rab8b		1.28	0.33	1.48	0.027*	1.16	0.42
	P36860	2	2	61.61	0.55	Ras-related protein Ral-B	Ralb		0.77	0.17	1.03	0.86	0.88	0.29
	D3Z8L7	2	2	68.82	0.43	Ras-related protein R-Ras	Rras		1.19	0.21	0.97	0.68	0.94	0.36
Lipid metabolism														
	Q06000	3	3	167.5	<0.001***	Lipoprotein lipase	Lpl	LPL	0.66	0.0088**	0.68	0.0046**	0.94	0.22
TCA cycle														
	Q8VHF5	14	14	678.33	0.17	Citrate synthase, mitochondrial	Cs	CS	1.01	0.80	1.06	0.13	1.11	0.048*
	P52873	45	44	3082.24	0.57	Pyruvate carboxylase	Pc	PC	1.01	0.87	1.03	0.68	1.05	0.091

Peptide information used for quantitation of these proteins is shown in S1 Table. Details include function, accession number, peptide count, and peptides used for quantitation, confidence score, *p*-value (ANOVA), description, gene name, trivial name, fold-change (LF-treated group/untreated control group), and *p*-value (Student *t* test).

*p < 0.05,

**p < 0.01,

***p < 0.001

ERK, extracellular signal-regulated kinase; HSL, hormone-sensitive lipase; LF, lactoferrin; PKA, protein kinase A; PLIN, perilipin.

### Activation of the cAMP signaling pathway

**Fig 1:**
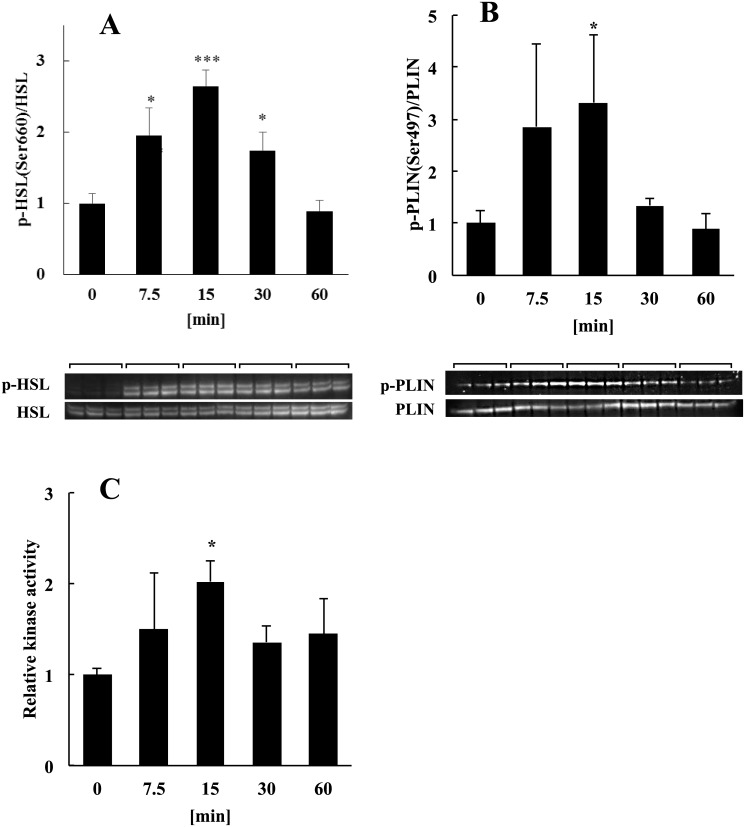
Analysis of the effects of LF on the phosphorylation of HSL and PLIN by PKA and determination of PKA activity. Phosphorylation of HSL and PLIN by PKA was detected in the presence or absence (0 min) of 1 mg/ml of LF. Phosphorylation levels normalized to protein expression levels of HSL and PLIN are shown. **(A)** Phosphorylation of HSL Ser660 and **(B)** PLIN Ser497 by PKA. **(C)** Analysis of PKA activity in adipocytes treated with LF. PKA activity in adipocytes was detected using an ELISA before (0 min) and after treatment with LF. Kinase activity normalized to the total protein determined by BCA is shown. The statistical significance of the data at each sampling time compared with the 0-min sample was evaluated using Dunnett’s multiple comparison test, and the data represent the mean ± SD values of triplicate determinations of one of three identical experiments. *p < 0.05, ***p < 0.001 HSL, hormone-sensitive lipase; LF, lactoferrin; PLIN, perilipin; PKA, protein kinase A; SD, standard deviation.

The lipolysis of lipid droplets requires the activation of HSL and perilipin (PLIN) [20] by PKA. To determine whether lipolysis occurred in adipocytes treated with LF, we performed western blot analysis to measure the phosphorylation of HSL and PLIN by PKA and determined the enzymatic activity of PKA using an ELISA. Recruitment of HSL to lipid droplets requires its phosphorylation by PKA on residues Ser659, Ser660, or both [20–22]. We found that HSL-Ser660 was phosphorylated within 15 min after the addition of LF (Fig 1A). Because there is no commercial anti-phospho-PLIN antibody suitable for western blot analysis, we used an antibody recommended for immunostaining analysis that recognizes the PKA phosphorylation site (Ser497) of human PLIN and crossreacts with mouse PLIN-Ser492. The amino acid sequences of mouse and rat PLIN are identical over a stretch of 20 amino acid residues encompassing Ser492. We found that this antibody detected the phosphorylation of rat PLIN-Ser492 within 15 min after the addition of LF to adipocytes (Fig 1B). Using an ELISA, we detected PKA activation 15 min after the cells were treated with LF (Fig 1C). These results suggest that treatment with LF activated PKA and that PKA phosphorylated HSL and PLIN within 15 min after treatment with LF.

### Activation of the ERK signaling pathway

**Fig 2:**
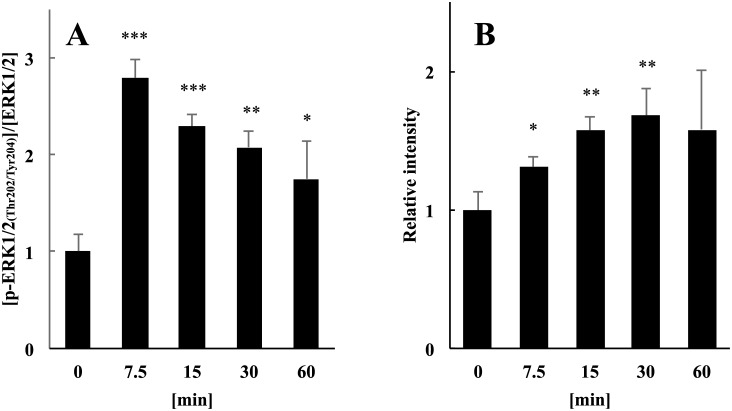
Analysis of the effects of LF on the activation of ERK1/2 and Ras. **(A)** Activation of ERK1/2 (Thr202/Tyr204) after treatment of adipocytes with LF. Phosphorylated ERK1/2 was detected in the presence or absence (0 min) of 1 mg/ml of LF. Phosphorylation levels normalized to protein expression levels of ERK1/2 are shown. **(B)** Ras activation through c-Raf in adipocytes treated with LF. Activated Ras captured from cell lysates using a pull-down assay kit (see Experimental Procedures) before (0 min) and after treatment with 1mg/ml of LF. Activated Ras eluted from the beads was detected using western blot analysis. Intensity levels normalized to the total protein determined by BCA. The statistical significance of the data at each sampling time compared with the 0-min sample was evaluated using Dunnett’s multiple comparison test, and the data represent the mean ± SD values of triplicate determinations of one of three identical experiments. *p < 0.05, **p < 0.01, ***p < 0.001. ERK, extracellular signal-regulated kinase; LF, lactoferrin; SD, standard deviation.

To determine whether LF activated Ras–Raf–ERK signaling (ERK signaling) in mature adipocytes, we determined the phosphorylation levels of ERK1/2 (Thr202/Tyr204). The ELISA results show that the phosphorylation levels of ERK1/2 peaked within 7.5 min after the addition of LF (Fig 2A), which is slightly earlier than the peak level observed for PKA activation (Fig 1C).

We next determined whether Ras proteins were activated in response to LF and then activated ERK1/2. We used an active Ras pull-down assay kit that only captured activated Ras through the Ras-binding domain of c-Raf. Using this technique, we found that after LF treatment, the amount of active Ras increased significantly within 7.5 min (Fig 2B). This finding indicates that Ras was activated within 7.5 min after the addition of LF and, in turn, activated c-Raf, leading to the phosphorylation of ERK1/2.

### Activation of cAMP response element binding protein

**Fig 3:**
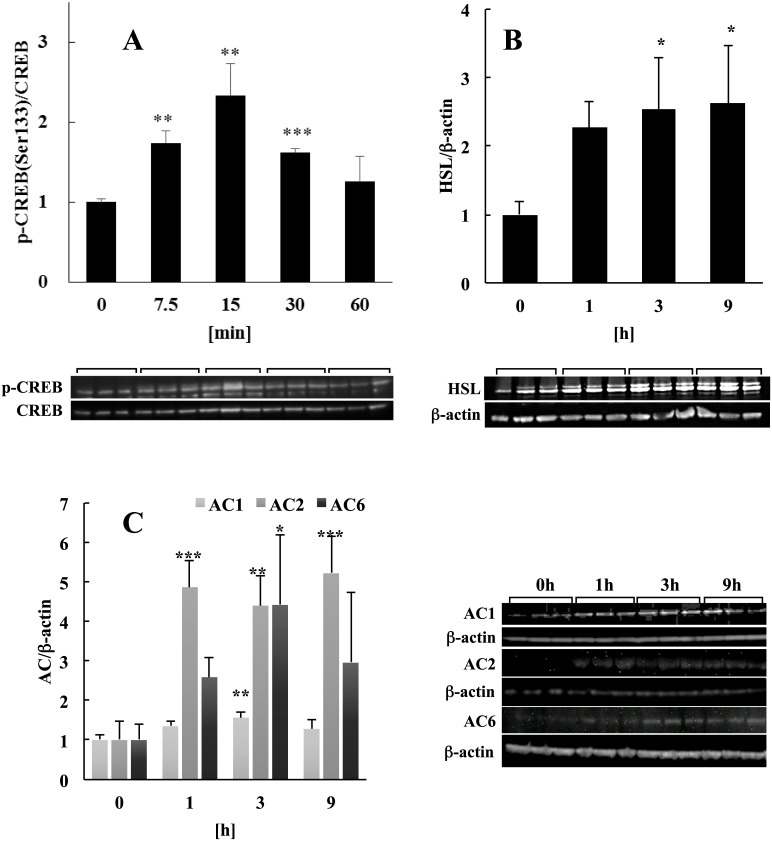
Analysis of the effect of LF on CREB activation. **(A)** Phosphorylation of CREB-Ser133 in adipocytes treated with LF. Phosphorylated CREB was detected in the presence or absence (0 min) of 1 mg/ml of LF. Phosphorylation levels normalized to the protein expression level of CREB. Changes in protein expression levels of **(B)** HSL and **(C)** AC isomers (AC1, 2, and 6) in the presence or absence (0 min) of 1 mg/ml LF normalized to the protein expression level of β-actin. The statistical significance of the data at each sampling time compared with the 0-min sample was evaluated using Dunnett’s multiple comparison test, and the data represent the mean ± SD values of triplicate determinations of one of three identical experiments. *p < 0.05, **p < 0.01, ***p < 0.001. AC, adenylyl cyclase; CREB, cAMP response element binding protein; HSL, hormone-sensitive lipase; LF, lactoferrin; SD, standard deviation.

cAMP response element binding (CREB) protein is a transcription factor that is activated by various extracellular stimuli and plays an important role in cell proliferation, differentiation, and survival [23]. CREB is most frequently activated by phosphorylation on Ser133 residue [24] by PKA [25]. Signaling via the ERK pathway activates CREB by phosphorylating CREB–Ser133 [17]. Since we have previously demonstrated that the cAMP and ERK signaling pathways were activated by LF in mature adipocytes, we reasoned that CREB was synergistically activated by these two pathways. As shown in Fig 3A, this assumption was confirmed by the detection of Ser133 phosphorylation within 7.5 min after the adipocytes were treated with LF.

### Analysis of proteins regulated by CREB

The changes in protein expression levels detected in our analyses described above may have been caused by transcription factors that act downstream in the cAMP and ERK signaling pathways. We focused on HSL because its 5′-UTR contains a long *cis*-acting regulatory element encompassing a CRE [26]. Moreover, our proteomic analysis revealed that HSL expression was upregulated in response to LF. Consistent with this result, western blot analysis revealed that the HSL expression level increased from 1 h and achieved a plateau after 3h by treatment with LF (Fig 3B).

In the cAMP signaling pathway, AC catalyzes the synthesis of cAMP. There are nine membrane-bound AC isoforms as well as a cytoplasmic isoform [27]. The detailed mechanisms of the transcriptional regulation of the genes encoding each isoform are unclear, although AC8 and AC6 have a CRE or CRE-like site in their 5′-UTRs [28, 29]. Therefore, we hypothesized that the transcription of the genes encoding the other AC isoforms are regulated by CREB. Western blot analysis revealed that the levels of AC1, 2, and 6, particularly that of AC2, were elevated from 1 h onward after LF treatment (Fig 3C).

**Fig 4:**
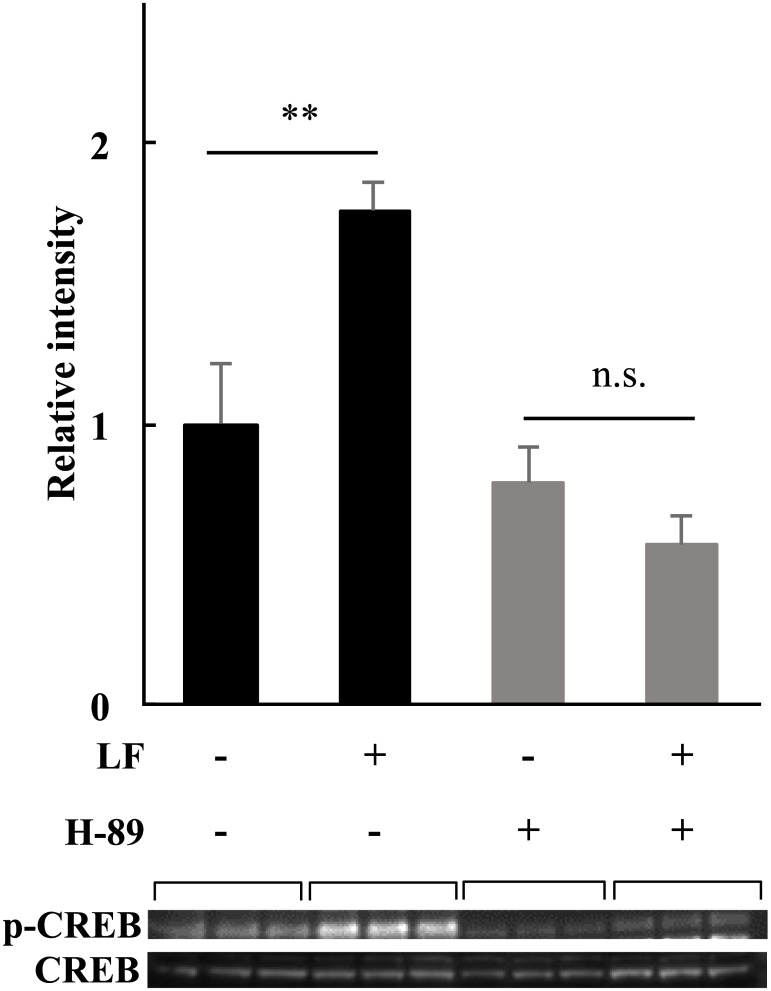
The influence of PKA activity inhibition on the downstream factor CREB. Phosphorylation level of CREB-Ser133 was detected 15 min after the treatment with 1 mg/ml of LF with or without H-89, a selective PKA inhibitor. Adipocytes were pre-incubated in H-89 starting at 30 min before the addition of LF. Phosphorylation level was normalized to the CREB protein expression level. The statistical significance of the differences in the data for LF treated vs. untreated samples was evaluated using the Student *t* test. **p < 0.01, n.s.; no significant difference. The data represent the mean ± SD of triplicate determinations of one of the three identical experiments. LF, lactoferrin; PKA, protein kinase A: CREB, cAMP response element binding protein: HSL, hormone sensitive lipase: SD, standard deviation.

In order to elucidate whether the change in the protein expression levels was a result of the early phosphorylation event of CREB, we used H-89, a selective inhibitor of cAMP-dependent protein kinase (PKA), to decrease the phosphorylation level of CREB. After 40 mM H-89 treatment, CREB was not phosphorylated 15 min after LF treatment (Fig 4).

**Fig 5:**
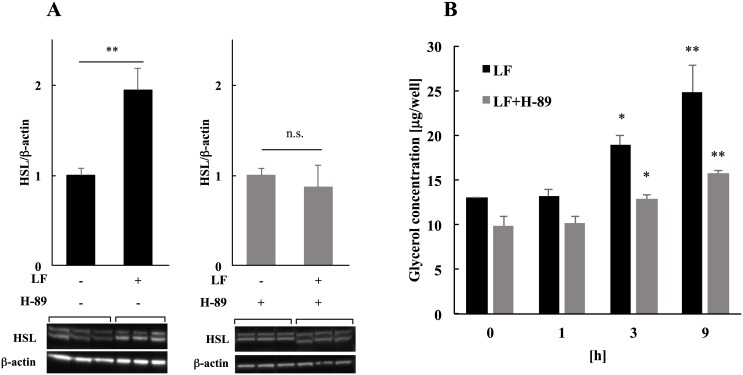
The influence of PKA activity inhibition on the HSL expression level and lipolysis by LF. **(A)** Change in expression levels of HSL by LF. The protein expression levels of HSL were detected 3 h after the treatment with 1 mg/ml of LF with or without H-89, a selective PKA inhibitor. Changes are normalized to the β-actin protein expression level. The statistical significance of the data compared with the LF untreated sample was evaluated using Student’s *t* test, and the data represent the mean ± SD values of triplicate determinations of one of the three identical experiments. *p < 0.05, **p < 0.01. **(B)** Activation of lipolysis by LF. The amount of glycerol in the medium was analyzed after the treatment with 1 mg/ml of LF with or without H-89, a selective PKA inhibitor, to quantify lipolysis. The statistical significance of the data compared with LF untreated cells was evaluated using Dunnett’s multiple comparison test. *p < 0.05, **p < 0.01. The data represent the mean ± SD values of triplicate determinations of one of three identical experiments. LF, lactoferrin; SD, standard deviation.

Three hours after the addition of LF, the expression level of HSL was detected with or without H-89. Thus, the expression level of HSL was not upregulated by LF in H-89 condition (Fig 5A). Moreover, we checked the time-dependent lipolysis level and found that H-89 treatment clearly decreased the lipolytic activity of LF but did not completely block it (Fig 5B). These results suggest that the early phosphorylation of transcription factors, such as CREB, could change the expression levels of proteins, such as HSL, and partly affect lipolytic action of LF.

### Evidence indicating that LRP1 is the LF receptor that mediates the induction of lipolysis in adipocytes

**Fig 6:**
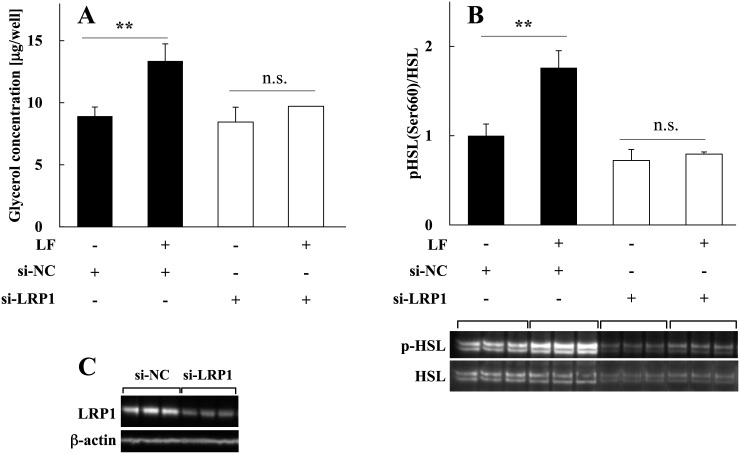
LF-induced lipolysis in LRP1-silenced adipocytes. **(A)** Activation of lipolysis by LF. To quantitate lipolysis, the amount of glycerol in the medium was analyzed 24 h after adding 1 mg/ml of LF. The statistical significance of the differences between LF treated and untreated cells was evaluated using the Student *t* test. **p < 0.01. The data represent the mean ± SD values of triplicate determinations of one of three identical experiments. **(B)** Activation of HSL by LF treatment. Phosphorylation of HSL was detected in the presence or absence of 1 mg/ml LF 15 min after the addition of LF. Phosphorylation levels normalized to protein expression levels are shown. The statistical significance was evaluated using the Student *t* test vs LF untreated control. **p < 0.01; n.s., no significant difference. The data represent the mean ± SD values of triplicate determinations of one of three identical experiments. **(C)** LRP1 silencing by siRNA. Adipocytes were transiently transfected with negative control siRNA (siNC) or LRP1 siRNA (siLRP1) (see Materials and methods). LRP1 protein expression was monitored by immunoblotting during each assay. Distinctive data is shown. β-actin was used as a loading control. HSL, hormone-sensitive lipase; LF, lactoferrin; LRP1, lipoprotein receptor-related protein 1; SD, standard deviation.

LF receptors expressed in various tissues mediate certain functions of LF [30]. However, the identities of LF receptors of adipocytes that mediate signaling by LF, particularly in lipolysis, are unknown. Because LRP1 is the LF receptor in various tissues, binds to multiple ligands, and mediates different functions [31], we hypothesized that it mediates lipolysis in adipocytes by binding to LF. To determine whether mature adipocytes require LRP1 to induce lipolysis by LF, LRP1 expression was silenced in rat mature adipocytes using an LRP1-specific siRNA (siLRP1). The lipolytic activity of transfected cells was analyzed by measuring glycerol production. We found that the lipolytic activities of LF were clearly attenuated in LRP1-silenced cells compared with cells transfected with negative control siRNA (siNC) (Fig 6A). Moreover, increased phosphorylation of HSL was undetectable in cells transfected with siLRP1 (Fig 6B). The silencing efficiency of LRP1 was 75%–80% (Fig 6C). These results suggest that LRP1 mediated the lipolytic signal delivered by LF to mature adipocytes.

**Fig 7:**
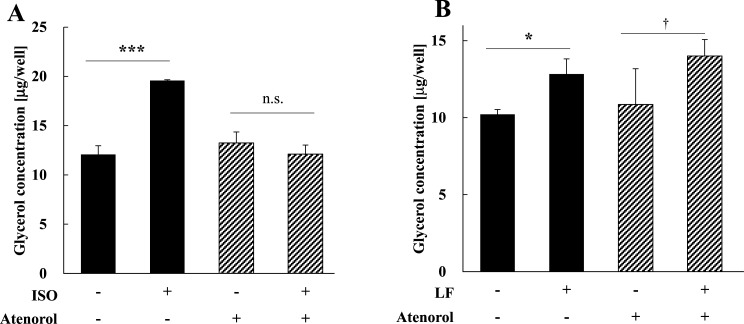
The influence of a βAR-blocker on the activities of lipolysis inducers. **(A)** Activation of lipolysis in adipocytes by ISO with or without a βAR inhibitor. The amount of glycerol in the medium was analyzed 24 h after adding 0.1 mM of ISO with or without atenolol (β_1_AR blocker) to quantitate lipolysis. The statistical significance of the differences in the data for LF treated vs. untreated cells was evaluated using the Student’s *t* test. ***p < 0.001, n.s.; no significant difference. The data represent the mean ± SD values of triplicate determinations of one of the three identical experiments. **(B)** Activation of lipolysis by LF with or without βAR inhibitor. The amount of glycerol in the medium was analyzed 24 h after adding 1 mg/ml of LF with or without atenolol (β_1_AR blocker) to quantify lipolysis. The statistical significance of the data of LF treated and untreated cells were evaluated using Student’s *t* test. *p < 0.05, ^†^ *p* < 0.1, n.s.; no significant difference. The data represent the mean ± SD values of triplicate determinations of one of the three identical experiments. LF, lactoferrin; SD, standard deviation.

Previous report by Goretzki et al. (1998) showed that LF binding to LRP1 increases the intracellular cAMP level and PKA activity via a stimulatory GTP-binding protein (Gα_s_) in the human melanoma cell line M21 that expresses LRP1 [32]. This result suggests the possibility that LRP1 activates the cAMP signaling pathway via interaction with Gα_s_. Therefore, we added atenolol, an inhibitor of β_1_AR and found that it inhibited the lipolytic action of the βAR agonist ISO but had no effect on lipolytic activity induced by LF (Fig 7A and 7B).

## Discussion

### Activation of the cAMP signaling pathway

AC is stimulated by a G protein-coupled receptor (GPCR) similar to the βAR, and HSL catalyzes a rate-limiting step in the pathway leading to lipolysis in adipocytes [33, 34]. In standard lipolysis, activated AC synthesizes cAMP, which activates PKA. Next, PKA phosphorylates and activates HSL. Activated HSL initiates fat breakdown at the surface of a lipid droplet [33, 34]. In the absence of lipolytic signaling, the surface of a lipid droplet is covered with the coating protein PLIN, which prevents lipolysis mediated by HSL. In contrast, once adipocytes receive lipolytic signals, PLIN is phosphorylated and activated by PKA and recruits HSL from the cytoplasm to the lipid droplet [20–22]. In the present study, we found that treating adipocytes with LF activated HSL, PLIN, and PKA, suggesting that LF activated the standard lipolytic pathway in mature adipocytes (Fig 1).

### Activation of the ERK signaling pathway

Ras-related proteins activate c-Raf (MAPKKK), a component of the classical MAPK pathway [35]. After its activation, c-Raf phosphorylates MEK1/2 (MAPKK), which in turn, phosphorylates and activates ERK1/2 (MAPK) [35]. Furthermore, signaling through this pathway induces various cellular responses by regulating the transcription of target genes [17]. Our data suggest that LF activates the ERK signaling pathway in mature adipocytes (Fig 2A and 2B). Consistent with these findings, previous studies in fibroblasts, osteoblasts, and keratinocytes have shown that LF phosphorylates and activates ERK1/2 to promote proliferation, migration, and differentiation of these cells as well as the induction of the contraction of the collagen matrix [36–38].

### Activation of CREB

CREB acts as a downstream transcription factor in the cAMP and ERK signaling pathways [17, 39]. We show here that treating adipocytes with LF activated CREB, upregulated HSL, and induced the synthesis of ACs (Fig 3A, 3B and 3C), which is consistent with the regulation by CREB of genes encoding HSL [26] and AC isoforms [28, 29]. Furthermore, the inhibition of PKA activity induced attenuated phosphorylation level of CREB (Fig 4) and resulted in blocking the upregulation of HSL by LF. Lipolytic activity by LF also decreased in this condition (Fig 5A and 5B). These findings suggest that LF activates several transcription factors as CREB via multiple signaling pathways in short-term, and these activation resulted in the alteration of the expression level of proteins related to lipolysis as HSL. Our lipolysis assay suggested that these changes could affect long-term lipolytic action of LF in part. Further investigation is required to clarify detailed mechanism of lipolysis induced by LF.

### Possible crosstalk between the cAMP and ERK signaling pathways

Multiple hormone receptors stimulate AC activity, primarily through interactions with G proteins [40]. Activation of protein tyrosine kinases (the predominant mechanism by which growth factors regulate intracellular functions) is viewed traditionally as a function of parallel and separate signaling cascades, but recent studies indicate that the activities of these pathways converge at multiple points [41, 42], particularly at the level of regulation of AC function [39, 43–47] For example, a Raf kinase (B-Raf or c-Raf) may mediate the phosphorylation of serine residues of AC [43, 44, 48]. Moreover, the mechanism of Raf kinase regulation is specific for different AC isoforms. For example, Ding *et*. *al*. (2004) demonstrated direct interactions between AC and Raf kinases using the human embryonic kidney cell line HEK293, suggesting an important but previously unrecognized interaction between these two key regulatory enzymes [48].

Multiple proteins regulate c-Raf activity, which are regulated by phosphorylation as well. For example, PKA inhibits c-Raf activity by phosphorylating c-Raf-Ser259 [49, 50]. PKA phosphorylation of certain GPCRs switches their signaling from G_s_-stimulation of AC to G_i_-coupled activation of ERK [51]. Future studies will be required to investigate the biological consequences of the interaction between cAMP and ERK signaling pathways in lipolysis.

### LRP1 may serve as an LF receptor in adipocytes

LF receptors in mammalian cells include intelectin, CD14, and LRP1 [30]. LRP1 is a multifunctional cell-surface receptor that belongs to the LDL receptor family and is expressed in several tissues [31]. Moreover, it comprises an 85-kDa membrane-bound C-terminal fragment and a noncovalently coupled 515-kDa N-terminal fragment. At least 40 different ligands bind to its extracellular domain [30]. The intracellular domain of LRP1 interacts with scaffolding and signaling proteins in a phosphorylation-dependent manner [52, 53] and functions as a coreceptor with other cell-surface or integral membrane proteins [31, 54, 55]. Thus, LRP1 is implicated in endocytosis and the regulation of signaling pathways, which play diverse biological roles in lipid metabolism, cell growth and differentiation, degradation of proteases, and tissue invasion [31].

In the present study, we found that LRP1-silenced adipocytes exhibited diminished lipolytic activity in the presence of LF. Further, LRP1 silencing inhibited HSL activation in adipocytes treated with LF, and it is also observed that the expression level of HSL apparently decreased in basal state of siLRP1 samples (Fig 6B). These results suggest that LRP1 needed to activate HSL sends lipolytic signaling, and induce lipolysis by LF. Furthermore, it is suggested that LRP1 is related in the regulation of HSL expression. Further studies are required to reveal basic transcriptional regulation of HSL in adipocytes.

Because previous report suggested the possibility that LRP1 activates the cAMP signaling pathway via interaction with Gα_s_, we inhibited β_1_AR and found that it had no effect on lipolytic activity induced by LF (Fig 7B). This result suggests that the activation mechanism of the cAMP signaling pathway of LF differs from that of the βAR-dependent GPCR signaling pathway at an upstream step of the pathway. Further studies are required to determine the mechanism of activation of ACs that act upstream of ACs in the LRP1 signaling pathway of adipocytes.

### A hypothetical model of signaling by LF in mature adipocytes

**Fig 8:**
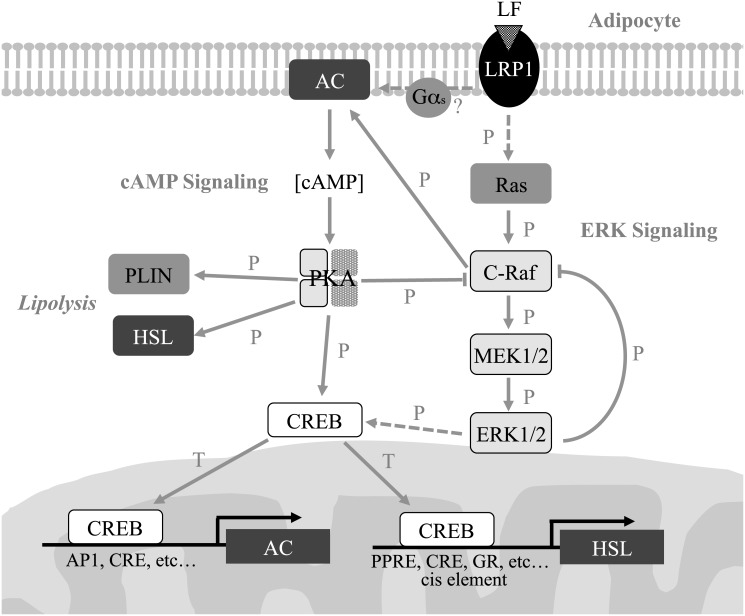
Hypothetical model depicting the induction of lipolysis by LF in mature adipocytes. T, Transcription; P, Phosphorylation; solid arrow, direct interaction; dashed arrow, indirect interaction. AC, adenylyl cyclase; CREB, cAMP response element binding protein; ERK, extracellular signal-regulated kinase; Gα_s_, G_s_ alpha subunit; HSL, hormone-sensitive lipase; LF, lactoferrin; LRP1, low-density lipoprotein receptor-related protein 1; PKA, protein kinase A; PLIN, perilipin.

The results of the present study led us to hypothesize that the induction of lipolysis in mature adipocytes by LF is caused via LRP1 signaling that is transmitted through the cAMP and ERK signaling pathways (Fig 8). This hypothesis differs from that proposed to explain GPCR-signaling induced by catecholamine receptors such as the βAR [40]. Here we show that activation of the ERK signaling pathway occurs within a few minutes after treatment of adipocytes with LF, which is followed by the indirect activation of PKA, CREB, HSL, and PLIN through cAMP production. There are many possibilities for interactions between the cAMP and ERK signaling pathways, such as direct interaction of AC and c-Raf. Moreover, c-Raf is stringently regulated by multiple proteins that are themselves regulated by phosphorylation, such as by PKA [49, 50]. Moreover, hyperphosphorylation of c-Raf by mitogen stimulation inhibits c-Raf activity [56]. Thus, the complex regulatory mechanism of the cAMP and ERK signaling pathways involves the initial reaction to LF stimuli, such as phosphorylation of ERK, HSL within 15 min that diminishes within 1 h. However, the activation of transcription factors such as CREB during this time rapidly induces the synthesis of HSL and AC, which could have a partial role in long-term lipid degradation. The metabolism of LF attenuates its signaling activity, returning adipocytes to their basal state. Further studies are required to establish the detailed mechanism of lipolysis induced by LF in adipocytes.

## Supporting Information

S1 Table Peptides used for quantitation of the proteins listed in Table 1. Details include function, charge, m/z, mass error (ppm), score, sequence, modifications, accession number, description, gene name, and *p*-value (ANOVA).
(XLSX)
